# The nomogram for predicting nasal bleeding after endoscopic transsphenoidal resection of pituitary adenomas: a retrospective study

**DOI:** 10.3389/fsurg.2024.1409298

**Published:** 2024-07-19

**Authors:** Ying Wang, Wei Wang, Qinghua Huang, Wei Yan, Meijuan Lan

**Affiliations:** ^1^ Nursing Department, The Second Affiliated Hospital of Zhejiang University School of Medicine, Hangzhou, Zhejiang Province, China; ^2^ Neurosurgery Department, The Second Affiliated Hospital of Zhejiang University School of Medicine, Hangzhou, Zhejiang Province, China

**Keywords:** neuro-oncology, pituitary adenomas, endoscopic transsphenoidal resection, nasal bleeding, nomogram

## Abstract

**Objective:**

This study aimed to develop and validate a dynamic nomogram to assess the risk of nasal bleeding after endoscopic transnasal transsphenoidal pituitary tumor resection.

**Methods:**

A retrospective analysis was conducted on patients who underwent endoscopic transnasal transsphenoidal pituitary tumor resection from June 2019 to June 2021. Univariate and multivariate logistic regression analyses were used to screen for independent risk factors for nasal bleeding from the training set. A multivariate logistic regression model was established, a nomogram was plotted, and it was validated in an internal validation set. The performance of the nomogram was evaluated based on the receiver operating characteristic (ROC) curve, calibration curve, and decision curve analysis (DCA).

**Results:**

The nomogram indicators included anticoagulant use, sphenoid sinus artery injury, nasal irrigation, platelet count (PLT), and constipation. The predictive model had an area under the ROC curve of 0.932 (95% CI: 0.873–0.990) and 0.969 (95% CI: 0.940–0.997) for the training and validation sets, respectively, indicating good discrimination. The calibration curve showed good consistency between the actual and predicted incidence of nasal bleeding (*p* > 0.05). DCA indicated that the nomogram had good clinical net benefit in predicting postoperative nasal bleeding in patients.

**Conclusion:**

In summary, this study explored the incidence and influencing factors of nasal bleeding after endoscopic transnasal transsphenoidal pituitary tumor resection and established a predictive model to assist clinical decision-making.

## 1 Introduction

Pituitary adenoma is one of the more common benign neuroendocrine tumors in clinical practice, accounting for about 10%–15% of intracranial tumors (1). Except for prolactinomas, about 96% of patients with pituitary adenomas can have their tumors removed surgically (2). Currently, endoscopic transnasal transsphenoidal surgery has become the preferred surgical procedure for the treatment of pituitary tumors. Compared with craniotomy for tumor removal, this procedure has the advantages of less trauma, clear vision, precise operation, and fast recovery (3).

At present, most doctors neglect the perioperative treatment of the nose during transnasal transsphenoidal pituitary tumor surgery, which may lead to complications such as nasal bleeding, sinusitis and nasal septal perforation (4). Delayed nasal bleeding is a well-known postoperative complication after pituitary adenoma resection. The incidence reported in recent series ranges from 0.6% to 3.3%. Most delayed nasal bleeding occurs 1–3 weeks after surgery, with a risk of bleeding 5–6 weeks postoperatively when the middle turbinate is removed (5).

Possible causes of nasal bleeding after pituitary tumor surgery include insufficient intraoperative hemostasis, severe mucosal injury, poor hemostatic effect of postoperative sponge tamponade, and a more dangerous cause that can easily cause massive bleeding is injury to the pterygopalatine artery or its posterior nasal septum branch in the lower corners of the bilateral sphenoid sinus (6). Postoperative nasal bleeding, due to its suddenness and large amount of bleeding, can easily block the airway and affect the patient's breathing, endangering the patient's life. Continuous bleeding can cause anemia and hemorrhagic shock; in severe cases, it can lead to secondary surgery for the patient, prolonged hospitalization period, and increased in-hospital mortality (7). Therefore, early identification of risk factors for nasal bleeding after endoscopic pituitary tumor surgery is of great clinical significance for the prevention and treatment of nasal bleeding.

A nomogram is a simple and easy-to-use graphical prediction tool that can be used to express the relationships between variables in a multifactorial regression analysis and to predict the probability of an outcome event (8). A nomogram can quickly calculate the likelihood of nasal bleeding in patients after endoscopic surgery based on the patient's individual characteristics and surgery-related factors, transforming complex regression equations into visualized graphics, making the results of the prediction model more readable and understandable (9). However, there is currently no research on establishing a predictive nomogram for nasal bleeding in patients after endoscopic pituitary tumor surgery.

The purpose of this study is to analyze the incidence and risk factors of nasal bleeding after endoscopic pituitary tumor surgery, propose feasible preventive measures based on relevant literature, and establish a nomogram to predict the risk of nasal bleeding in patients after surgery, with the aim of providing a reference for the prevention and care of postoperative nasal bleeding complications in future patients.

## 2 Methods

### 2.1 Study cohort

Patients who underwent endoscopic transsphenoidal pituitary tumor resection from June 2019 to June 2021 were included as the study objects. Inclusion criteria: (1) Meeting the diagnostic criteria of pituitary tumor; (2) Endoscopic transsphenoidal pituitary tumor resection during hospitalization; (3) Informed consent and willingness to participate in the study; (4) 80 years old ≥18 years old. Exclusion criteria: (1) quit the researcher midway; (2) Patients with incomplete clinical data. This study has been approved by the hospital Ethics Committee [(2021) No. 0287].

### 2.2 Patient management

All patients were managed by a multidisciplinary team of experienced neurosurgeons, endocrinologists, neurosurgery residents, and physician assistants. Intraoperatively, the skull base was closed in various ways depending on the complexity and size of the defect. This included simple closures with dural grafts and multilayer complex closures achieved through the use of abdominal fat packing and tensor fascia lata grafts. All patients were managed with standardized institutional practices, including standing checks for sodium balance and strict charting for volume status.

### 2.3 Data collection

Baseline data of patients, including gender, age, marriage, education, job, medical treatment, BMI, smoking, alcohol consumption and work type, were retrospectively collected. And disease history (diagnosis of disease, history of hypertension, history of diabetes, history of nasal surgery, history of nasal disease, history of constipation), history of medication (preoperative hormone therapy, taking anticoagulant drugs), factors related to surgery (operation time, intraoperative sphenoid artery injury, postoperative dilatant sponge tamponade, nasal tamponade material, tamponade method, time of pulling out the tamponade, etc.), blood-related indicators [white Cell, red blood cell, platelet, prothrombin time (PT), activated partial thrombin time (APTT)] and postoperative nasal irrigation.

### 2.4 Statistical analysis and validation of the nomogram

Continuous variables were compared by *t*-test and categorical variables were compared using the χ^2^ test or Fisher's exact test. The presence of multicollinearity between covariates was determined by the variance inflation factor (VIF). Univariate logistic analysis was used to assess the association between covariates and outcomes. Variables with *p* < 0.05 were included in the multifactorial regression. Patients were randomly divided into a training cohort and a validation cohort. Univariate and multivariate logistic regression analyses were performed. The performance of the nomogram was verified in the validation set using the following methods: area under the curve (AUC) of the receiver operating characteristic (ROC) curve, calibration plots and Hosmer-Lemeshow goodness-of-fit tests (HL tests), and decision curve analysis (DCA). *p* < 0.05 was considered to be statistically significant. All statistical analyses were performed with R statistical software (V.4.2.1).

## 3 Results

### 3.1 Baseline characteristics

A total of 412 eligible patients, who were included in the study, were randomly divided into a training cohort (*n* = 206) and a validation cohort (*n* = 206). There were 16 (7.73%) patients with nosebleed in the training set and 22 (10.68%) in the validation set. Detailed characteristics for these two cohorts showed no difference (*P* > 0.05) and demonstrated homogeneity between two cohorts (Table 1).

**Table 1:** Characteristics at baseline between training set and validation set.

Variables	Validation set *N* = 206	Training set *N* = 206	*P*-value
Female	110 (53.14)	97 (47.09)	0.219
Age, years	50.91 ± 12.84	50.70 ± 14.29	0.873
BMI	24.33 ± 3.33	24.13 ± 3.42	0.556
Diabetes	22 (10.63)	18 (8.74)	0.516
Hyperlipidemia	13 (6.28)	13 (6.31)	0.99
Hypertension	56 (27.05)	56 (27.18)	0.976
Drinking	68 (32.85)	64 (31.07)	0.698
Smoking	68 (32.85)	49 (23.79)	0.052
Anticoagulant	4 (1.93)	5 (2.43)	0.731
Constipation	46 (22.22)	46 (22.33)	0.979
Nasal bleeding	16 (7.73)	22 (10.68)	0.300
Spongy distention	179 (86.47)	174 (84.47)	0.563
Sphenoid artery injury	11 (5.31)	19 (9.22)	0.126
Operation duration	103.23 ± 53.94	109.59 ± 55.79	0.240
Preoperative hormone	40 (19.32)	39 (18.93)	0.919
Bilateral nostrils	112 (54.11)	98 (47.57)	0.184
Nasal wash	157 (75.85)	145 (70.39)	0.211
Functional pituitary adenoma	97 (46.86)	111 (53.88)	0.153
Invasive pituitary adenoma	59 (28.50)	57 (27.67)	0.851
WBC, 10^9^/L	9.55 ± 4.89	9.05 ± 4.50	0.280
RBC, 10^12^/L	4.14 ± 0.89	6.74 ± 32.09	0.247
PLT, 10^9^/L	241.79 ± 85.88	232.65 ± 67.20	0.578
PT, s	13.25 ± 0.81	13.21 ± 0.82	0.623
APTT, s	36.14 ± 4.12	37.07 ± 4.97	0.066

BMI, body mass index; WBC, white blood cell count; RBC, red blood cell count; PLT, platelet count; PT, prothrombin time; APTT, activated partial thromboplastin time.

In the training set, the patients with epistaxis were more likely to have History of hyperlipidemia (18.2% vs. 4.89%, *P* = 0.015), anticoagulant drug use (13.6% vs. 1.09%, *P* = 0.000) and constipation (81.8% vs. 15.2%, *P* = 0.000), as compared to the control group. In terms of therapeutic measures received, Patients with nosebleed had more intraoperative sphenoid artery injury (27.3% vs. 7.1%, *P* = 0.002), bilateral nostrils (27.2% vs. 55.4%, *P* = 0.012), preoperative hormone (40.91% vs. 16.30%, *P* = 0.005), operation duration (128.68 ± 61.71 vs. 104.29 ± 53.73, *P* = 0.006), nasal wash (18.18% vs. 76.63%, *P* = 0.000), invasive pituitary adenoma (50.00% vs. 25.00%, *P* = 0.013) and PLT (271.82 ± 85.96 vs. 212.64 ± 66.78, *P* = 0.005) (Table 2).

**Table 2:** Characteristics at baseline between nosebleed and nomal groups in the training set.

Variables	Nosebleed *N* = 22	Nomal *N* = 184	*P*
Female	9 (40.91)	88 (47.83)	0.539
Age	49.36 ± 16.85	50.86 ± 14.00	0.644
BMI	23.82 ± 3.00	24.17 ± 3.47	0.650
Diabetes	2 (9.09)	16 (8.70)	0.951
Hyperlipidemia	4 (18.18)	9 (4.89)	0.015*
Hypertension	6 (27.27)	50 (27.17)	0.992
Drinking	6 (27.27)	58 (31.52)	0.684
Smoking	3 (13.64)	46 (25.00)	0.237
Anticoagulant	3 (13.64)	2 (1.09)	0.000**
Constipation	18 (81.82)	28 (15.22)	0.000**
Spongy distention	19 (86.36)	155 (84.24)	0.795
Sphenoid artery injury	6 (27.27)	13 (7.07)	0.002**
Preoperative hormone	9 (40.91)	30 (16.30)	0.005**
Operation duration	128.68 ± 61.71	104.29 ± 53.73	0.006**
Bilateral nostrils	6 (27.27)	102 (55.43)	0.012*
Nasal wash	4 (18.18)	141 (76.63)	0.000**
Functional pituitary adenoma	8 (36.36)	103 (55.98)	0.081
Invasive pituitary adenoma	11 (50.00)	46 (25.00)	0.013*
WBC, 10^9^/L	9.85 ± 4.53	8.96 ± 4.50	0.381
RBC, 10^12^/L	3.67 ± 0.72	7.11 ± 33.95	0.636
PLT, 10^9^/L	271.82 ± 85.96	212.64 ± 66.78	0.005**
PT, s	13.50 ± 1.18	13.18 ± 0.77	0.080
APTT, s	37.12 ± 4.25	37.06 ± 5.05	0.955

BMI, body mass index; WBC, white blood cell count; RBC, red blood cell count; PLT, platelet count; PT, prothrombin time; APTT, activated partial thromboplastin time.

* *P* < 0.05.

** *P* < 0.01.

### 3.2 Logistic and variables selection

In order to identify the risk factors of epistaxis, the variables included in the nomogram model were screened out, and the relevant variables were screened by stepwise regression. The test of multicollinearity of the model found that the VIF values in the model were all less than 5, which meant that there was no collinearity problem. It can be seen that we finally included five indicators, all independent predictors of epistaxis, which were anticoagulant (OR = 12.244, 95% CI 11.471–17.464, *p* = 0.006), sphenoid artery injury (OR = 22.608, 95% CI 2.521–37.535, *p* = 0.007), nasal wash (OR = 0.010, 95% CI 0.001–0.096, *p* = 0.000), PLT (OR = 1.020, 95% CI 1.009–1.031, *p* = 0.000), and constipation (OR = 44.301, 95% CI 10.950–81.085, *p* = 0.000) (Table 3).

**Table 3:** Logistic regression analysis of candidate factors of nosebleed in the training cohort.

	OR	95% CI	*P*	VIF
Anticoagulant	12.244	11.471–17.464	0.006*	1.057
Constipation	44.301	10.950–81.085	0.000**	1.023
Sphenoid artery injury	22.608	2.521–37.535	0.007*	1.035
Nasal wash	0.010	0.001–0.096	0.000**	1.011
PLT	1.020	1.009–1.031	0.000**	1.085

PLT, platelet count; PT, prothrombin time; APTT, activated partial thromboplastin time.

* *P* < 0.05.

** *P* < 0.01.

### 3.3 Nomogram construction and validation

**Figure 1:**
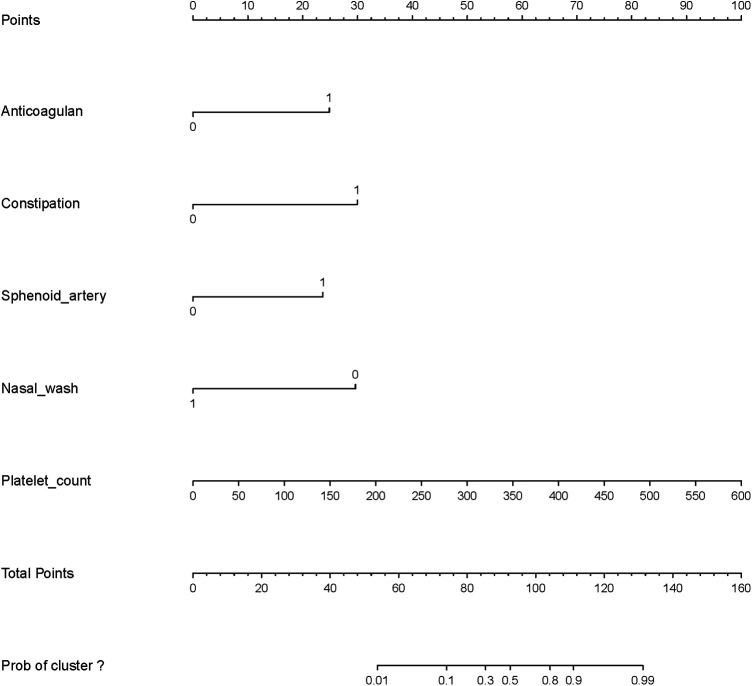
Nomogram to identify the risk of nosebleed in patients after pituitary tumor surgery, based on logistic regression analysis. To acquire the corresponding scores for each variable, draw a vertical line upward to the “Points” axis. Sum the score for all predictors and locate the final value on the “Total Points” axis. Draw a line straight down to the “Risk” axis to determine the risk of nosebleed.

The multivariate model was visualized as a nomogram (Figure 1). To apply the nomogram, users should draw a virtual vertical line from each variable to the “Points” axis to identify the points attributed by each variable. Then, users need to compare the summed points with the bottom scale to assess the probability of nosebleed.

**Figure 2:**
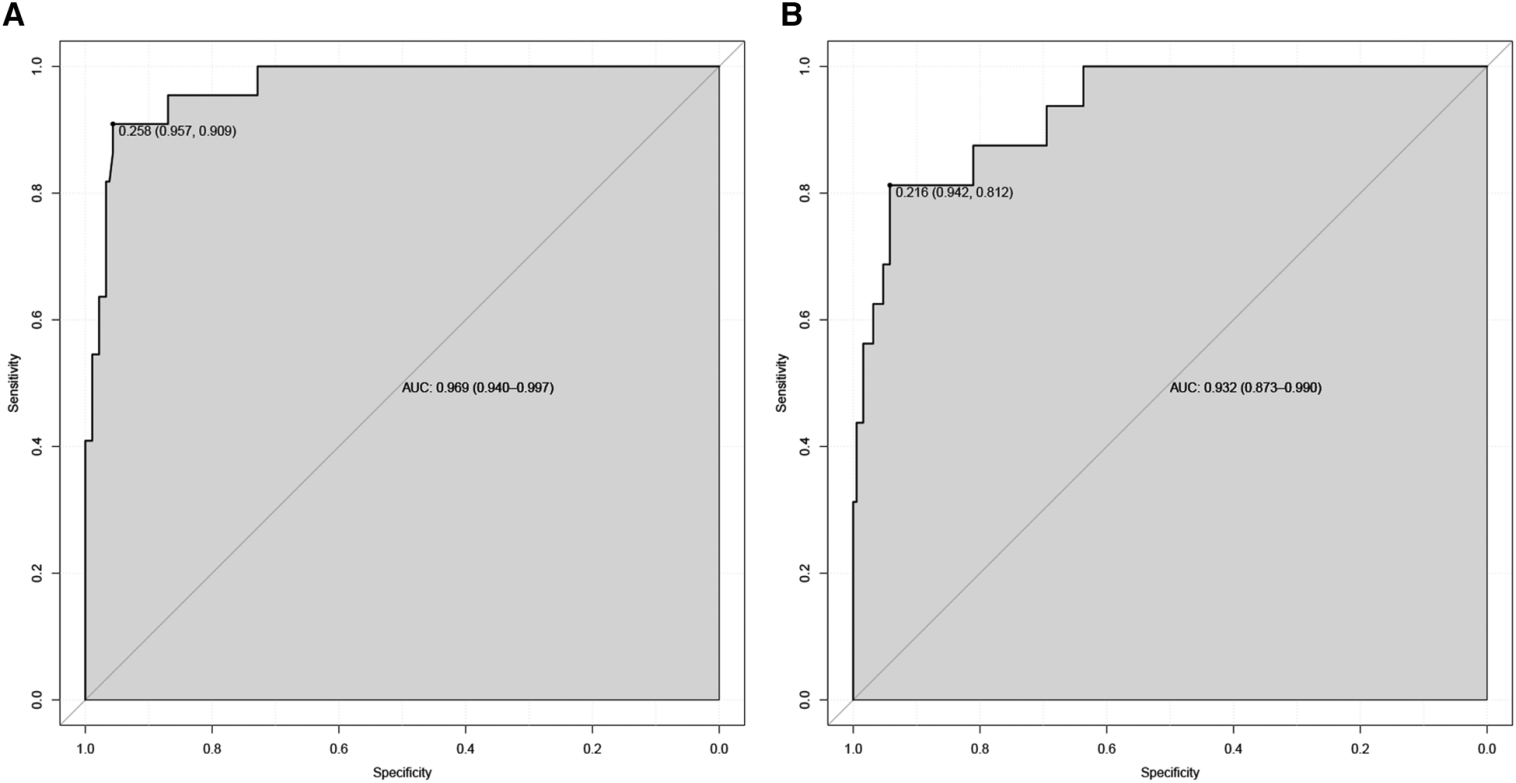
Receiver operating characteristic curve of the nomogram. The an area under the curve (AUC) of the nomogram for the prediction of nosebleed in patients was 0.969 in the training set (**A**) and 0.932 in the validation set (**B**).

**Figure 3:**
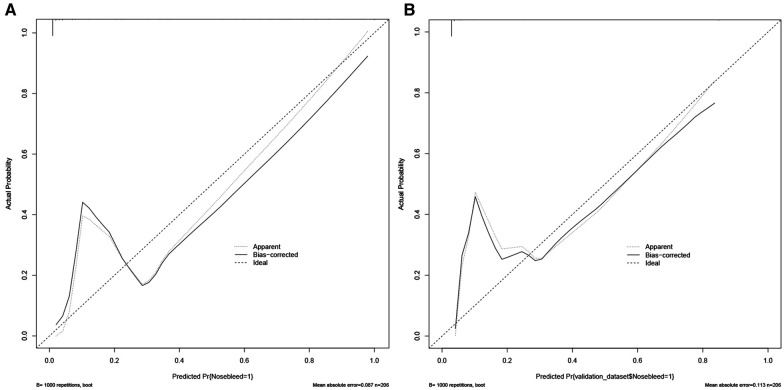
Calibration curves of the predicted nomogram in the training set (**A**) and validation set (**B**) results of the Hosmer-Lemeshow test demonstrate that the *p*-value of the training set is 0.8814 and the validation set is 0.4835, respectively.

Nomograms showed good differentiable value for nosebleed in patients after pituitary tumor surgery. And differentiation was assessed by area under the curve (AUC) in the receiver operating characteristic curve (Figure 2). Internally verified training AUC = 0.969 (95% CI: 0.940–0.997) and external validation AUC = 0.932 (95% CI: 0.873–0.990). Calibration curve was also generated, which reflected adequate prediction accuracy using the nomogram mode (Figure 3). The horizontal axis is the predicted possibility, and the vertical axis is the actual possibility, since the closer the curve is to the dashed line of the diagonal, the better the calibration.

**Figure 4:**
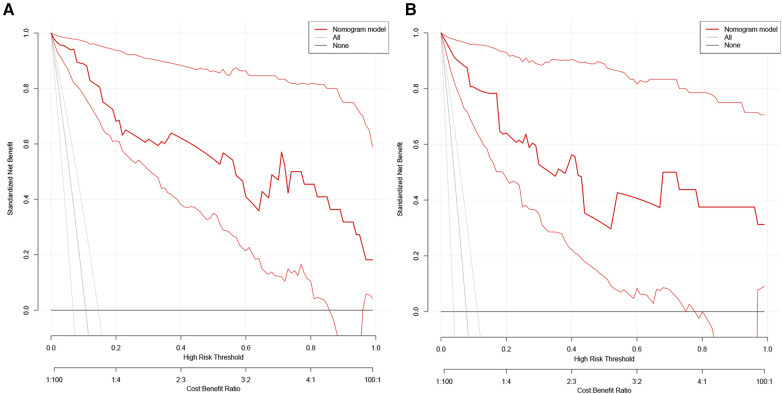
Decision curve analysis (DCA) of the nomogram in the training set (**A**) and the validation set (**B**).

Decision curve analysis (DCA) of the nomogram were in the Figure 4. The horizontal axis indicates that no one received the intervention, and the net gain was 0. The diagonal line indicates that everyone received the intervention. The smaller the threshold, the greater the net gain. Interventions that avoided curves show a higher net reduction in interventions per 100 patients as the prediction threshold increases in the range of 0.01–1 in both the training (Figure 4A) and validation sets (Figure 4B). The DCA and interventions that avoided curves visualize that the nomogram has significant predictive value.

## 4 Discussion

This study shows that anticoagulant, sphenoid artery injury, nasal wash, PLT, and constipation are independent risk factors for nasal bleeding in patients after pituitary tumor surgery. These factors were included in the nomogram model, which was tested and verified to have high accuracy and reliability. The nomogram developed in this study is feasible and stable for predicting postoperative nasal bleeding in patients and can assist neurosurgical medical staff in providing individualized postoperative nasal bleeding risk assessment and guidance for pituitary tumor patients after surgery.

Pituitary tumor is a common intracranial benign tumor. At present, endoscopic transnasal transsphenoidal surgery is mainly used for resection (10). This procedure has the advantages of small trauma, fast recovery, clear vision, precise operation, etc., which can reduce the damage to the skull base structure and the incidence of intracranial infection (11). However, the complex anatomy of the nasal sphenoid sinus, including redundant mucosa, high vascularity, fragile mucosa and complex sphenoid sinus septum, makes endoscopic transsphenoidal surgery challenging and prone to postoperative nasal bleeding (12). Patients who are discharged from the hospital and suddenly experience nasal bleeding outside the hospital will cause fear and anxiety in patients, affecting postoperative respiratory and olfactory functions, increasing the risk of infection and reoperation. Improper handling of massive bleeding can also lead to anemia and hypoxemia, endangering life (13). Most bleeding can be controlled by conservative measures such as local cauterization, hemostatic drugs and nasal packing. Uncontrollable and/or massive nasal bleeding is potentially fatal and requires timely diagnosis and treatment (14).

There have been few studies on nasal bleeding after pituitary tumor surgery. One retrospective study analyzed 330 patients who underwent endonasal endoscopic surgery, with a total of 14 nasal bleeding events. Most events were controlled by simple packing, and 5 events were controlled by surgery. This study suggests that smoking, being male, older age, and having hypertension are potential risk factors for nasal bleeding (15). The results of this study show that a history of anticoagulant use, constipation, injury to the pterygopalatine artery, nasal wash, and platelets are major risk factors for nasal bleeding after endoscopic transsphenoidal pituitary tumor surgery.

Patients taking anticoagulants are more likely to bleed, which is easy to understand. The common complication of using anticoagulants is bleeding. It may be that anticoagulants increase the risk of bleeding by antagonizing clotting factors or dissolving fibrinogen (16), and the risk of postoperative nasal bleeding in preoperative anticoagulant patients is greatly increased. Platelets participate in physiological hemostasis in the human body through adhesion, aggregation, release and contraction functions and maintain the integrity of the vascular wall. The normal platelet count is 100–300 × 10^9^/L. When the platelet count drops below 50,000, patients with minor trauma may experience bleeding points (17). Surgery and trauma can cause platelets to increase reactively, often within the normal range (18). The platelets of the patients included in this study were all within the normal range, and the platelet count of patients with nasal bleeding was significantly increased. Perhaps this is because the patient's coagulation function increased reactively. Constipated patients are prone to cardiovascular diseases such as hypertension. Straining during defecation can increase abdominal pressure and thus increase intracranial pressure. Therefore, constipated patients are prone to rupture and bleeding from increased pressure on the pterygopalatine artery or its branches (19).

In addition to the patient's own factors that increase the risk of nasal bleeding, neurosurgeons should pay attention to the details during surgery to reduce complications such as nasal bleeding. Intraoperative nasal irrigation is a protective factor for postoperative nasal bleeding. Studies have shown that postoperative nasal irrigation can effectively improve complications such as nasal bleeding, nasal adhesion, and olfactory dysfunction in patients (20). Nasal irrigation can promote the movement of nasal mucosal cilia, increase the rate of cilia elimination of secretions and inflammatory factors, reduce nasal mucosal dryness, congestion and edema, thereby reducing the risk of nasal bleeding (21). Due to the lack of three-dimensional visual depth in neuroendoscopy, vascular injury is easily caused during operation (22). When the tumor texture is soft or the high intrasellar pressure caused by the occupation, the tumor wall collapses rapidly when the capsule is cut open. Small perforating arteries are easily ruptured after being pulled. The most commonly injured blood vessels are the pterygopalatine artery or its posterior septal branch in the lower lateral corner of the sphenoid sinus. The branches of the pterygopalatine artery mainly supply the anterior lower part of the nasal septum, and most of the nasal bleeding occurs in the easy-to-bleed area in the anterior lower part of the nasal septum (23). Therefore, neurosurgeons should operate finely during surgery, with the endoscope lens facing the surgical field, try not to pull on the tumor body, and indirectly operate on tumors that are in close contact with the sellar diaphragm.

A nomogram was constructed using the five factors of anticoagulant, sphenoid artery injury, nasal wash, PLT, and constipation. This is the first nomogram to predict postoperative nasal bleeding in patients with pituitary tumors. It performed well in terms of predictive performance, calibration and clinical application, providing valuable information for different patients to choose individualized treatment plans. The model has the advantages of being simple, intuitive, easy to operate and apply. Compared with traditional statistical methods, it can better reflect the individual differences and complexity of patients. It may provide personalized risk assessment of postoperative nasal bleeding for patients, thereby helping clinicians to formulate reasonable surgical plans, preventive measures and follow-up strategies, improve surgical safety and effectiveness, reduce the incidence of complications and medical costs. This study is a single-center retrospective study with a relatively small sample size. There may be selection bias and insufficient representation of the entire population. In addition, this study did not consider factors such as the patient's genes, immunity, and endocrine system, which may also be related to postoperative nasal bleeding. Therefore, further verification and optimization of this model is needed in larger-scale and multi-center cohorts.

## 5 Conclusion

The results showed that anticoagulant use, sphenoid artery injury, nasal wash, platelet count (PLT), and constipation were independent risk factors for nasal bleeding after surgery. A nomogram was constructed using these five factors to predict the risk of postoperative nasal bleeding in patients. The nomogram performed well in terms of predictive performance, calibration and clinical application.

## Data Availability

The raw data supporting the conclusions of this article will be made available by the authors, without undue reservation.
